# Structural brain changes in the development of essential tremor: toward early diagnosis

**DOI:** 10.3389/fnagi.2026.1767582

**Published:** 2026-07-16

**Authors:** Karmele Lopez-de-Ipina, Jose Ignacio Sánchez-Méndez, Jordi Solé-Casals, Rafael Romero-Garcia, Elsa Fernandez, Anujan Poologaindran, Maite García-Sebastian, Pablo Martinez-Lague, Catalina Requejo, J.F. Martí-Massó, Alberto Bergareche, John Suckling

**Affiliations:** 1 Department of Psychiatry, University of Cambridge, Cambridge, United Kingdom; 2 EleKin Research Group, System Engineering and Automation Department, University of the Basque Country UPV/EHU, Donostia-San Sebastian, Spain; 3 NTT DATA Europe and LATAM, Arlington, VA, United States; 4 Data and Signal Processing Research Group, University of Vic-Central University of Catalonia, Barcelona, Spain; 5 Instituto de Biomedicina de Sevilla (IBiS) HUVR/CSIC/Universidad de Sevilla/CIBERSAM, ISCIII, Dpto. de Fisiología Médica y Biofísica, Sevilla, Spain; 6 The Alan Turing Institute, British Library, London, United Kingdom; 7 CITA Alzheimer Foundation, Donostia-San Sebastián, Spain; 8 Departamento de Ciencias Médicas Básicas, Instituto de Medicina Molecular Aplicada (IMMA) Nemesio Díez, Facultad de Medicina, Universidad San Pablo-CEU, CEU Universities, Madrid, Spain; 9 Neurodegenerative Disorders Area, Biodonostia Health Research Institute, Donostia-San Sebastián, Spain; 10 Movement Disorders Unit, Department of Neurology, University Hospital Donostia, San Sebastián, Spain; 11 Biomedical Research Networking Centre Consortium for the Area of Neurodegenerative Diseases (CIBERNED), Madrid, Spain

**Keywords:** brain circuits and networks, degenerative disease, diagnosis, essential tremor severity, neuroimaging

## Abstract

**Background:**

Essential tremor (ET) is the most common movement disorder. Its etiology and neuropathology remain controversial, but could be clarified with neuroimaging.

**Objectives:**

This study investigated brain structures derived from T1-weighted MRI associated with tremor severity in analyses focusing on the motor circuit and whole-brain to test competing hypotheses.

**Methods:**

Structural MRI at 3T was acquired from 50 ET participants and 50 healthy controls (HC). Voxel-based morphometry and surface-based processing generated measures of brain structure. Three two-group comparisons were undertaken: 1) HC versus ET participants; 2) HC versus ET participants with low tremor; and 3) HC versus ET participants with high tremor. For ET participants, correlations with tremor severity were also explored.

**Results:**

Within the motor circuit and across the whole brain, no significant case–control differences were observed after correction. Likewise, vertex-wise analyses of cortical volume, cortical thickness, and gyrification did not reveal significant differences. Uncorrected voxel-wise analyses suggested subtle reductions in the cerebellum and the motor circuit, particularly in ET participants with greater tremor severity. However, these effects were generally small and not consistently observed across analyses.

**Conclusion:**

The present analysis showed that ET severity was associated with weak structural differences, mainly within the motor circuit, although these findings did not survive correction. Consistent with previous literature, structural MRI abnormalities in ET appeared subtle and heterogeneous across studies, potentially becoming more detectable in patients with more severe symptoms. Although not designed to assess disease progression directly and noting that no finding survived correction for multiple comparisons, the direction of uncorrected exploratory trends within the motor circuit is consistent with the hypothesis of progressive structural alterations; however, this interpretation remains speculative and requires replication in larger, longitudinal cohorts.

## 1 Introduction

Essential tremor (ET) is among the most common adult-onset movement disorders worldwide. Recent meta-analysis of 42 population-based studies estimated a pooled all-age prevalence of approximately 1.33%, with a median crude prevalence of 0.4% and a mean of 0.67%. Prevalence increases sharply with age: in individuals aged 65 years or above, pooled prevalence reaches 5.79% (95% CI 4.14–8.05%), with descriptive analyses reporting median crude prevalence up to 5.9–8.0% and even higher rates in the oldest age groups. In many epidemiologic reports, no significant difference in prevalence by sex has been found (*p* = 0.90) (Louis and Ferreira, 2010; Louis and McCreary, 2021). While PD typically presents with a resting tremor, ET presents with a low-amplitude upper extremity tremor during purposeful movement. The neuropathology of ET is not well understood. There is an underlying neurodegenerative process of the cerebellum in ET, but its origin remains controversial (Jellinger, 2014; Benito-León, 2014). In fact, epidemiologic and neuropathologic studies have reported a link between PD and ET since a subtype of ET could be prone to develop PD, but not vice versa, by spreading of Lewy body pathology found in the brainstem (Louis and Ottman, 2013; Yoo et al., 2023). Moreover, this ET-PD subtype supposes a different subclass of PD with preceding ET that could support an understanding of the neurodegenerative origins of ET (Yoo et al., 2023). Therefore, exploring in detail the ET subtypes and the homologies and differences with respect to PD may lead to a better prognostic accuracy.

The consensus statement by the Movement Disorder Society on the classification of tremor (Bhatia et al., 2018) defines ET as an isolated tremor syndrome of bilateral, upper limb action tremor of at least 3 years duration, with or without tremor in other locations (e.g., head, voice, or lower limbs (Avecillas-Chasin et al., 2019)), in the absence of other neurological signs such as dystonia, ataxia, or parkinsonism. However, there is evidence that ET is phenotypically heterogeneous (Louis, 2018; Bolton et al., 2023; Tsuboi et al., 2025). Thus, tremor classification was revised to include a new term, Essential Tremor plus (ET-plus), as a separate entity from ET and characterized by the presence of neurological signs other than action tremor with additional neurological signs of uncertain clinical significance, such as impaired tandem gait, dystonic posturing, memory impairment, or other mild neurological signs of unknown clinical significance that do not suffice to make an additional syndrome classification or diagnosis. According to some authors (Louis et al., 2020), the use of the term ET plus has potential implications for research because it will complicate efforts to assess the incidence and prevalence of ET, will make it challenging to do both longitudinal cohort and genetic studies in the presence of phenotypic heterogeneity within families, and will complicate therapeutic trials. Cases exhibiting additional “soft” neurological signs (e.g., impaired tandem gait, questionable dystonia, mild cognitive changes, and rest tremor) are designated as ET plus, rather than simply ET (Ortega-Robles and Arias-Carrión, 2025, 2025, 2025). The ET-plus classification remains controversial. A detailed post-mortem study comparing cerebellar pathology in “pure” ET versus ET-plus found no significant differences across 13 of 14 quantitative metrics; after correction for multiple comparisons, none of the metrics distinguished ET from ET-plus, challenging the notion that they are distinct clinicopathological entities. Thus, it is currently unclear whether ET-plus represents a separate disorder, a subtype, or merely a stage in a broader ET spectrum (Gionco et al., 2021). Ideally, analytical subgroups should be defined by their genetic, physiological bases, or therapeutic response profile, but that is not possible yet, and so other biomarkers, such as those based on neuroimaging analysis, have significant potential (Tuite and Dagher, 2013; Sharifi et al., 2014; Klaming and Annese, 2014; Mueller et al., 2017; Sepúlveda Soto and Fasano, 2020).

The Central Nervous System (CNS) architecture, partly organized in loops of reciprocal inhibition as well as the existence of excitatory connections both at the neuronal and inter-neuronal network levels, makes it prone to produce aberrant oscillatory activities (Shaikh et al., 2008). In this paradigm, ET is the manifestation of the dysfunction of multiple tremorigenic pathways, sometimes functionally overlapping, in which their normal oscillatory function, often in the motor circuit, is increased and/or pathologically deregulated and expressed clinically as a tremor (Brittain and Brown, 2013; Hallett, 1998; Tremor, 2014). Although the exact function of these oscillations is still a matter of debate, they seem to play an important role in central motor control in humans (Kristeva et al., 2007), where cortical (somatomotor and motor cortex) and subcortical areas are involved.

The participation of cerebellar–thalamus and olive-cerebellar pathways have been discussed in the prior literature (Raethjen and Deuschl, 2012), but the origin of the oscillatory activity and the basis of the transition of physiological oscillations that occur during normal motor control to pathological tremor remain unknown (Lenka and Jankovic, 2021).

There are currently two competing hypotheses to explain the pathophysiological mechanisms of ET: (i) ET is due to a generalized physiological alteration of neuronal function (e.g., neuronal oscillations) that produces tremor and other subtle alterations of neural functions; and (ii) the symptoms and signs of essential tremor are a consequence of a selective and primary neurodegeneration to a “motor circuit” of ET comprising the cerebellum, thalamus, and basal ganglia, as well as motor and premotor cortices.

To clarify this controversy, this study was structured to investigate both pathways involved in the genesis of tremor and the topography of anatomical lesions associated with ET. Irrespective of the specific mechanism, it was hypothesized that there are differences in the motor system of ET patients that would be related to the degree of affliction of the disease. In this context, the present study was designed to investigate neuroanatomical differences in the motor system associated with ET. Specifically, we evaluated grey matter volume, cortical thickness, cortical volume, and local gyrification index (LGI) across groups: healthy controls, ET patients with low tremor severity or intensity, and ET patients with high tremor severity. We hypothesized that structural changes in cortical, subcortical, and cerebellar regions would correlate with symptom severity, reflecting either changes in a distributed tremorgenic network or compensatory remodeling of network architecture.

Statistical testing was undertaken using estimates of grey matter (GM), cortical volume (CV), cortical thickness (CT), and local gyrification Index (LGI), a measure inspired by the classical two-dimensional gyrification index (Zilles et al., 1988). To test the first hypothesis, case–control differences in brain structure were analyzed to identify which, if any, brain circuits were affected by ET dependent on symptom severity. Then, within-patient relationships to tremor severity were tested within the cortical, subcortical, and cerebellar areas of the putative ET circuit. Whole-brain testing was also undertaken to identify generalized changes in anatomy. The design was based on three groups: a control (unaffected) group and an ET group, divided into two sub-groups with low and high tremor severity.

## 2 Materials and methods

### 2.1 Participants

Fifty patients with ET and 50 unaffected healthy controls (HC) were recruited from a descriptive observational cohort of familial and sporadic ET cases carried out in the Movement Disorders Unit at the Donostia University Hospital, San Sebastian, Spain, from January 2015 to June 2017. The cohort included both familial and sporadic presentations of ET, and family history status was recorded for all participants as part of the broader parent study. The present neuroimaging subsample comprises patients with both familial and sporadic ET, as the two subgroups did not differ significantly in tremor severity, age, or other key demographic characteristics. No *a priori* neuroimaging hypothesis was formulated to distinguish familial from sporadic cases in this structural MRI analysis. All participants were initially diagnosed by a movement disorder specialist based on the established clinical criteria (Bhatia et al., 2018). Historical data, such as age, age at onset, gender, handedness, disease duration, and clinical symptoms, were collected using a standard questionnaire. All participants in this study underwent a series of structured questionnaires and a comprehensive neurological and neuropsychological assessment conducted by three experienced movement disorder specialists. Each patient received a diagnosis of ET after the first evaluation, which was subsequently confirmed by consensus with the clinical team based on a review of the available data and electrophysiological records from the second evaluation using the established diagnostic criteria. The Fahn–Tolosa–Marin rating scale score (Fahn et al., 1993) (TRS) was conducted to assess the tremor severity of patients with ET. The scale consists of three parts: TRS, part A, B, and C. The TRS parts A and B were combined as TRS-A and B to obtain a single score, and it was used to evaluate tremor severity and to define low and high tremor groups. The TRS-C was assessed by self-evaluation to record the quality of life of ET participants. Seventeen ET participants were screened for cognitive performance with the Montreal Cognitive Assessment (MoCA) (Nasreddine et al., 2005). HC were not cognitively screened. All ET participants were drug-naïve.

Exclusion criteria for all ET participants were as follows: an isolated moderate–severe head tremor, parkinsonism, ataxia, orthostatic hypotension, gaze palsy, and a secondary cause of tremor. Healthy controls were excluded if they had any neurologic illness or family history of ET after clinical evaluation and review of medical records (Hale et al., 2020; Louis et al., 2010).

After being given a complete description of the study, all participants provided written and verbal informed consent prior to any procedures. The study was approved by the Ethics Committee of the Hospital Donostia (Comité Ético de Investigación, Clínica del Área Sanitaria de Gipuzkoa. Acta n° 4/2010).

### 2.2 Magnetic resonance imaging acquisition

Imaging data were acquired from all participants with a 3T MRI scanner (Magnetom Trio Tim, Siemens Medical Systems, Germany) at the Centro de Investigación y Terapias Avanzadas (CITA) Alzheimer Foundation. This system used an image matrix coil with 32 radio frequency channels, providing high image quality with integrated parallel acquisition. High-resolution T1-weighted images were acquired with the MPRAGE 3D sequence with the following parameters: repetition time, TR = 2,300 ms; echo time, TE = 30 ms; inversion time, TI = 900 ms; field-of-view, FOV = 244 × 244 mm^2^; 1-mm isotropic voxels.

### 2.3 Structural image processing

Estimates of GM volume were made at each intracerebral voxel using (FSLVBM, n.d.; Douaud et al., 2007; Good et al., 2001; Smith et al., 2004; Andersson and Smith, 2007)^,^ an optimized VBM protocol (Good et al., 2001) carried out with FSL tools (FSLVBM, n.d.; Smith et al., 2004). Images were also processed by FreeSurfer to estimate regional CT, CV, and LGI from three-dimensional cortical surface models derived using intensity and continuity information (Fischl and Dale, 2000).

In more detail, structural MRIs were brain-extracted and GM-segmented before being registered to the Montreal Neurological Institute (MNI) 152 standard space using non-linear registration (Andersson and Smith, 2007). The resulting images were averaged and reflected along the x-axis to create a left–right symmetric, study-specific GM template. Native GM images were then non-linearly registered to this study-specific template and “modulated” to correct for local expansion (or contraction) due to the non-linear component of the spatial transformation. The modulated GM images were then smoothed with an isotropic Gaussian kernel of sigma 3 mm.

Region of interest (ROI) analyses were undertaken with the Desikan-Killiany atlas (Desikan et al., 2006), consisting of 308 regions, with each region of approximately equal size (500 mm^2^). The atlas was applied using the standard (FreeSurferWiki, n.d.) template (fsaverage) and a backtracking algorithm that subdivided the regions such that the final regions were constrained by the original anatomical boundaries (Romero-Garcia et al., 2012). The parcellation of the regions was spatially mapped from the standard stereotactic coordinate system of the MNI space to an individual’s MPRAGE acquisition space using surface-based markers. This approach provides better alignment of cortical landmarks than volume-based registration. Moreover, registering to acquisition space does not result in an age-associated bias, making it feasible to accurately compare structural properties and patterns (Ghosh et al., 2010).

Head motion was calculated by FreeSurfer’s Euler number, which measures the quality of surface reconstruction (FreeSurferWiki, n.d.; Rosen et al., 2018), and between-group testing was undertaken.

### 2.4 Statistical design

The study’s statistical design consisted of three case–control comparisons: 1. the HC group versus all participants with ET; 2. HC versus ET patients with low (TRS-A&B < =11) tremor: low tremor group (LTG); 3. HC versus ET patients with high (TRS-A&B > 11) tremor: high tremor group (HTG).

To test the hypothesis that ET is a consequence of a selective and primary dysfunction, between-group structural changes were tested in a motor circuit constructed with regions corresponding to cerebellum, motor and premotor cortices, thalamus, and basal ganglia. Testing was undertaken using the total GM volumes, CT, CV, and LGI from within the motor circuit as a single region. Age, sex, and intracranial volume (ICV) were added as covariates. Inference was by permutation test (*t*-test, 1,500 randomizations) with *p*-values corrected for multiple comparisons for the number of tests applied (Permutation Test, 2025) and regression models (Vul and Pashler, 2012). Group effects, such as age, sex, and intracranial volume (ICV), were assessed as covariates. Interaction terms between group and age (or tremor severity) were included to evaluate differential trajectories between groups. Group effects were also assessed using generalized linear models (GLMs), with groups included as the main factor and age, sex, TRS, and intracranial volume (ICV) entered as covariates. GLM analyses were implemented in Python. False discovery rate (FDR) correction was applied. Significance was set at *p* < 0.05, corrected. To test the hypothesis of a generalized alteration in brain structure, between-group tests of parenchymal volume, total GM, CT, CV, and LGI were performed with age, sex, and ICV as covariates.

Maps of GM volume, CT, CV, and LGI were spatially tested for between-group differences in a voxel-wise approach. Between-group inference on GM volume was carried out with a general linear model (GLM), including a covariate for ICV. Inference was with the randomized (FSL) permutation-based non-parametric statistical test with a threshold-free cluster enhancement (TFCE) (FSLVBM, n.d.; Smith and Nichols, 2009; Vul and Pashler, 2012) and a false discovery rate (FDR), corrected for multiple comparisons using 15,000 permutations with significance set at *p* < 0.05. Significant clusters were identified, and average data were extracted and labeled with AtlasQuery and autoaq using the standard atlases of FSL (Douaud et al., 2007; Smith et al., 2004).

Between-group vertex-wise analyses of FreeSurfer-derived metrics were tested with a GLM at each surface vertex, again covarying for ICV. The Different Onset, Same Slope (or DOSS) option was used along with a Clusterwise correction for Multiple Comparisons (FreeSurferWiki, n.d.; Vul and Pashler, 2012). A multiple comparisons correction was applied using a permutation inference with 15,000 permutations implemented in the Permutation Analysis of Linear Models (PALM) software (Winkler et al., 2015; Winkler et al., 2016; Winkler et al., 2016). An additional conservative Bonferroni correction was applied across the number of surface vertices tested, providing family-wise error rate control complementary to the FDR approach used in the ROI analyses. This dual-correction strategy was chosen to ensure robustness of any positive finding, given the exploratory nature of the whole-brain vertex-wise approach. Significance was set at *p* < 0.05, corrected.

A within-group design was also tested, including patients with ET only. The relationships of tremor severity, as a continuous measure of the TRS-A&B, and GM volume, CV, CT, and LGI were tested by partial correlation covarying for age, sex, and ICV for the motor circuit and whole brain values. Significance was set at *p* < 0.05.

## 3 Results

### 3.1 Demographic and clinical characteristics

**Figure 1:**
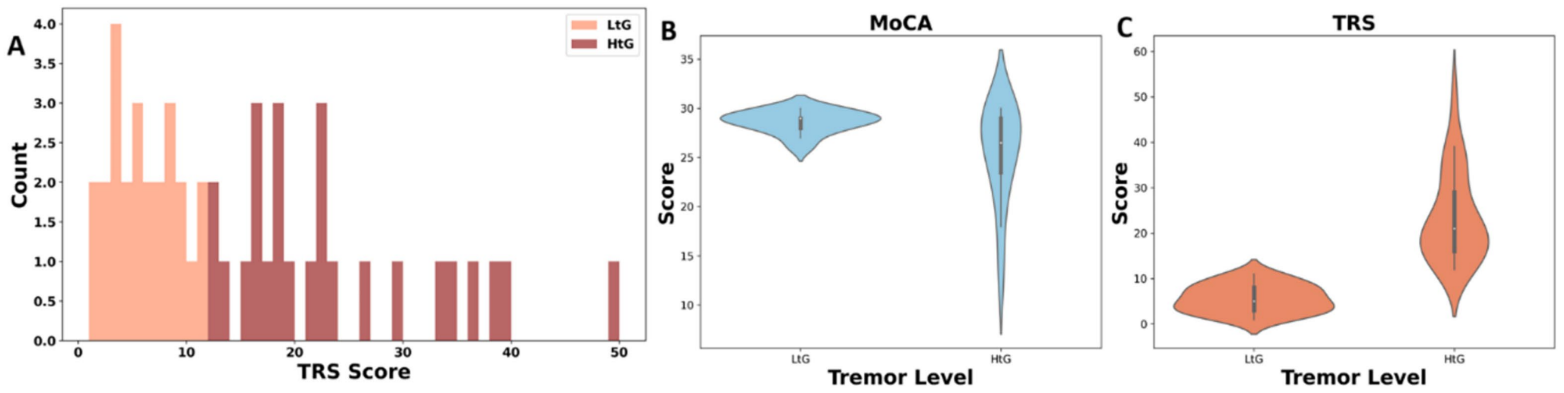
**(A)** TRS-A&B scores for ET participants. **(B)** Montreal cognitive assessment (MoCA) for LTG and HTG groups, and **(C)** TRS-A&B scores for LTG and ETG groups, with a threshold between the groups of 11.

There were 50 participants recruited into the HC group (mean age = 60.04 ± 13.73 years, *N* = 25 female, range = 60–93 years). All HC participants had TRS-A&B tremor level = 0. Fifty participants were recruited with ET (mean age = 61.6 ± 15.80 years, *N* = 25 female; range = 25–86 years) having a range of 1–50 of tremor severities. ET participants were stratified into LTG (TRS-A&B < =11) and HTG (TRS-A&B > 11) (Torii et al., 2022). Figure 1. MoCA was collected from seven participants in the LTG and ten in the HTG. Demographic, MoCA, and TRS-A&B data are summarized in Table 1 and Figure 1.

**Table 1:** Demographic data for healthy control (HC) participants and patients with essential tremor (ET), classified according to TRS-A&B tremor severity into low tremor group (LTG) and high tremor group (HTG).

Group	HC (*N* = 50)	ET (*n* = 50)	LTG (*n* = 25)	HTG (*n* = 25)
Age	60.20 ± 11.73	60.90 ± 16.21	52.60 ± 16.40	69.20 ± 11.19
Gender (M: F)	25:25	25:25	16:09	9:16
Tremor level (TRS-A&B)	0 ± 0	14.50 ± 11.51	5.64 ± 3.05	23.4 ± 9.85
Euler number	26.92 ± 13.28	37.10 ± 25.72	26.32 ± 13.57	48.81 ± 31.47
Montreal cognitive assessment (MoCA)		27.07 ± 3.93 (*n* = 17)	28.64 ± 1.15 (*n* = 7)	25.50 ± 5.05 (*n* = 10)

No significant differences were found in age, gender, ICV, or Euler number in the HC versus ET comparisons. There was a significant difference in the HC versus LTG comparison for age (*p* = 0.024, *t* = 2.18), but not for ICV, gender, or Euler number, and in the HC versus HTG comparison for age (*p* = 0.003, *t* = −3.18) and Euler number (*p* = 0.002, *t* = −3.11), but not for ICV or gender.

**Figure 2:**
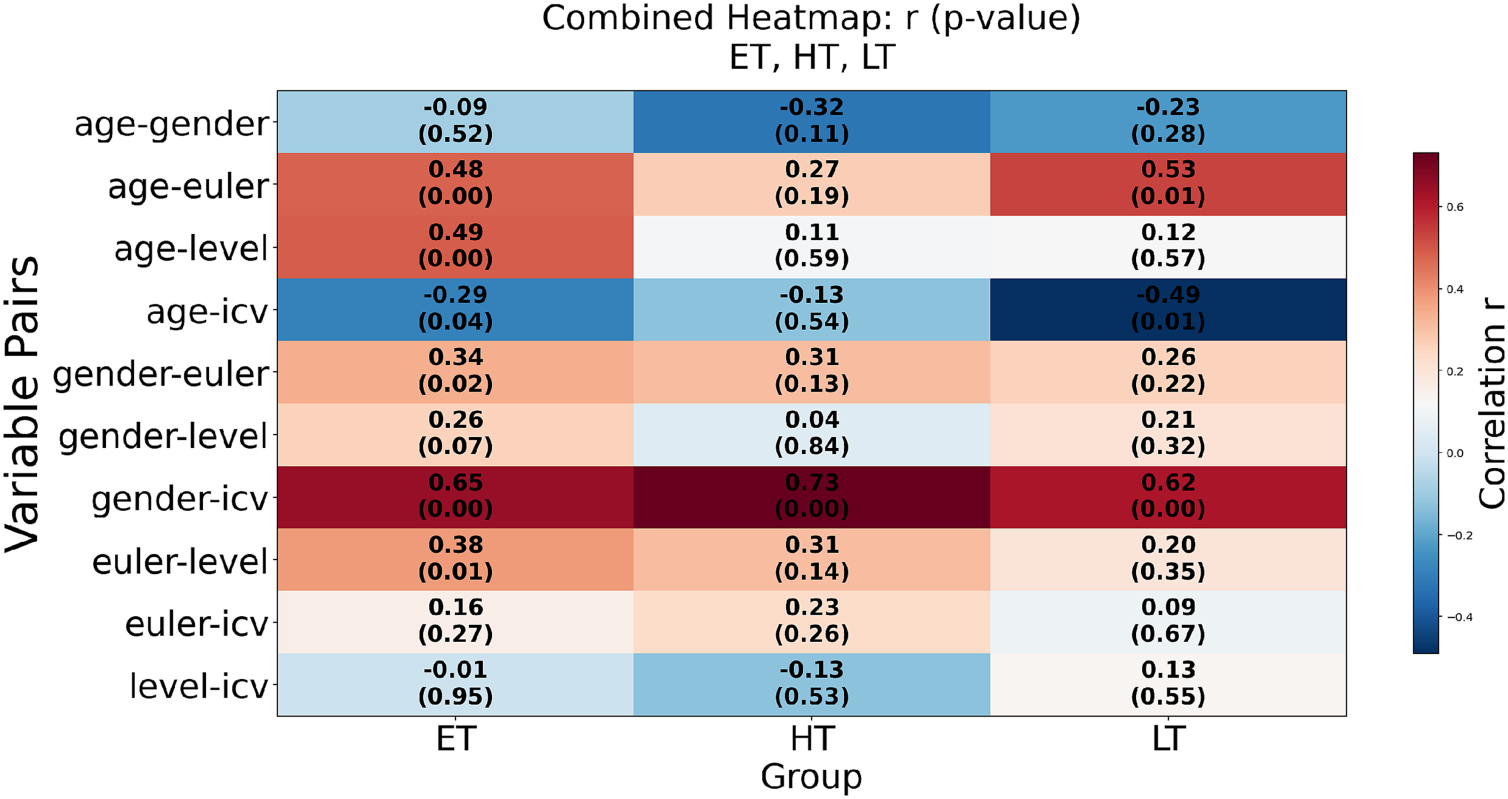
Combined heatmap: *r* (*p*-value) for correlations among variables.

**Figure 3:**
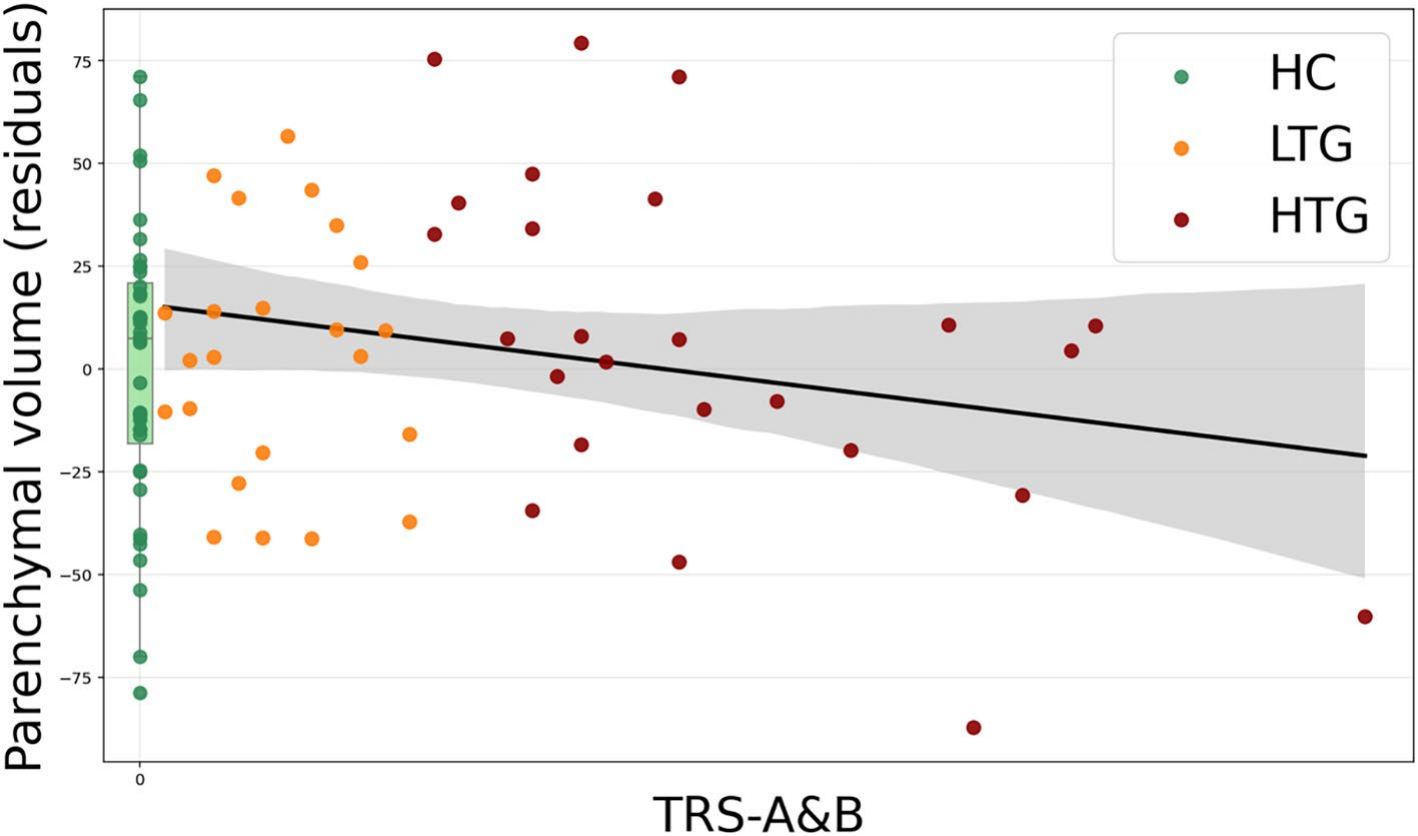
Parenchymal volume within the ET group partial correlations for parenchymal volume, with TRS-A&B controlling for age, sex and ICV (residuals).

There were significant correlations between Euler number and age (*p* = 0.0003, *r* = 0.48) and tremor level and age (*p* = 0.0009, *r* = 0.49) across ET patients. There was a significant relationship between Euler number and age in the LTG group (*p* = 0.006, *r* = 0.53), but no significant relationship between gender or ICV and tremor level (Figure 2). All covariates, such as age, sex, and ICV showed acceptable multicollinearity, with variance inflation factors (VIFs) remaining below 2, Figure 3.

### 3.2 Between-group differences in brain structure

**Figure 4:**
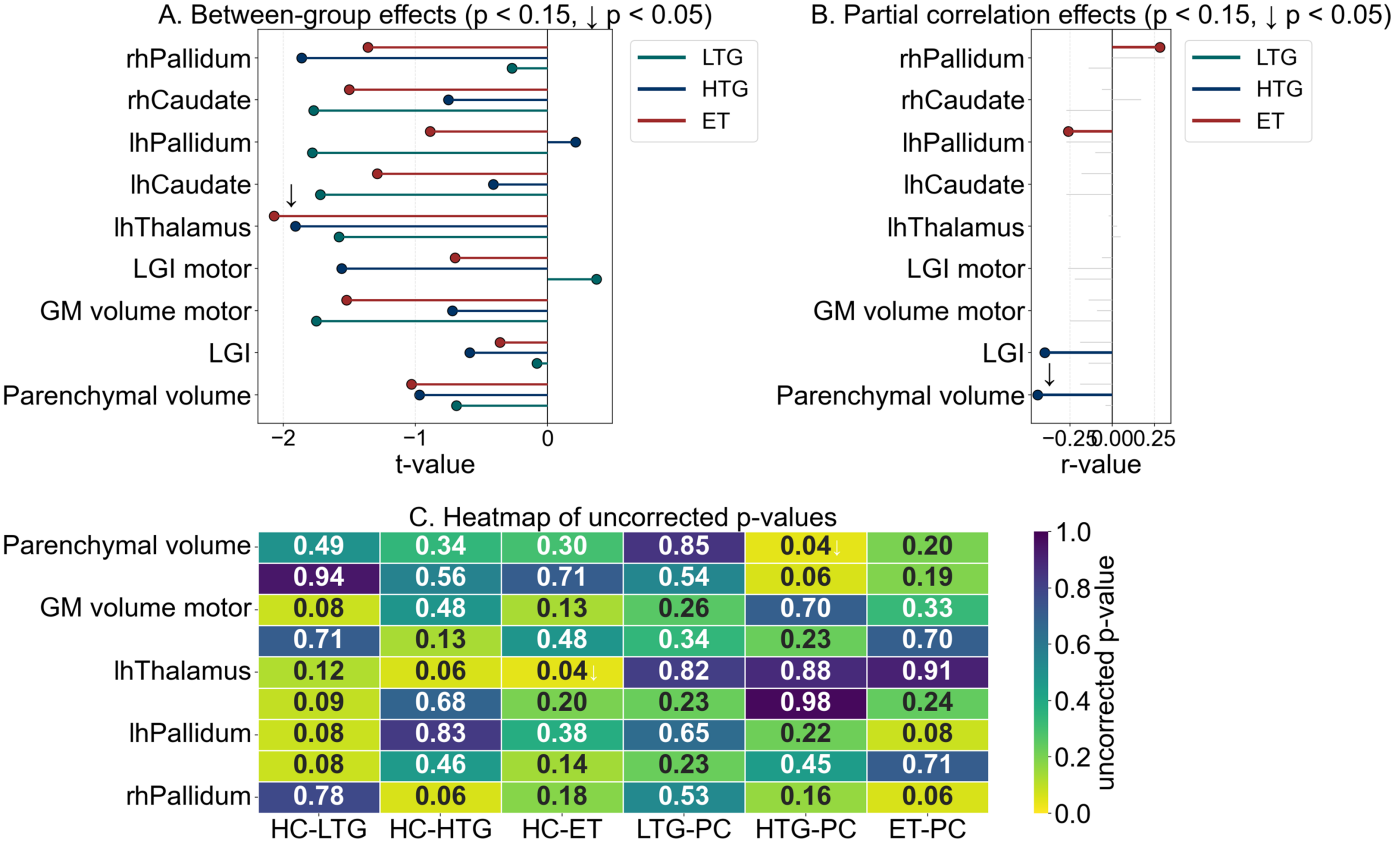
**(A)** Between-group comparisons (HC vs LTG, HC vs HTG and HC vs ET) are shown as t-values across cortical, motor, and subcortical regions. All uncorrected effects with *p* < 0.15 are displayed, with nominally significant uncorrected effects indicated (↓ *p* < 0.05, uncorrected). **(B)** Partial correlations between regional measures and tremor severity are shown as r-values, with nominally significant uncorrected effects indicated (↓ *p* < 0.05, uncorrected). Findings indicate a distributed pattern of subcortical involvement, with subtle effects in thalamic ↓ and basal ganglia regions and a global structural association in Parenchymal volume ↓ and LGI. Note: no effect survived FDR correction for multiple comparisons. These are exploratory observations only. **(C)** Heatmap of uncorrected *p*-values.

Mean values of imaging-derived measures with standard deviations are reported in Tables 2, 3, and the results of between-group comparisons are presented in Tables 4, 5. In uncorrected analyses, no between-group differences reached conventional significance (*p* < 0.05, uncorrected) for whole-brain or motor circuit measures of total GM volume, cortical thickness (CT), cortical volume (CV), or total local gyrification index (LGI). At the subcortical level, a nominally significant uncorrected effect was observed for HC versus ET in the left thalamus (*p* = 0.04, uncorrected, *t* = −2.07), with sub-threshold trend-level uncorrected effects in HC versus LTG (*p* = 0.12, uncorrected, *t* = −1.58) and HC versus HTG (*p* = 0.06, uncorrected, *t* = −1.91). Additional exploratory, uncorrected effects were observed in basal ganglia structures, such as the left caudate (HC vs. LTG: *p* = 0.09, uncorrected, *t* = −1.72), left pallidum (HC vs. LTG: *p* = 0.08, uncorrected, *t* = −1.78), right caudate (HC vs. LTG: *p* = 0.08, uncorrected, *t* = −1.77), and right pallidum (HC vs. HTG: *p* = 0.06, uncorrected, *t* = −1.86). A weak effect was also observed for in-motor GM volume in HC versus LTG (*p* = 0.08, uncorrected, *t* = −1.75). Although preliminary, these findings may suggest subtle involvement of basal ganglia–thalamic circuits in specific groups (Figure 4). Consistently, GLM results indicated a subtle but non-significant group effect in the left thalamus for HC versus ET. However, none of the reported effects survived FDR correction (pFDR). No corrected *p*-values reached statistical significance in these analyses (all FDR-corrected *p*-values > 0.05). Overall, these results highlight exploratory, uncorrected trends within motor-related networks, without evidence of robust effects after correction.

**Table 2:** Volume means and standard deviations of whole brain and motor circuit imaging-derived measures for each group.

	HC	ET	LTG	HTG
Whole brain				
ICV (cm^3^)	1465.02 ± 130.79	1495.92 ± 155.19	1494.73 ± 156.52	1497.11 ± 153.87
Parenchymal vol. (cm^3^)	1078.41 ± 98.43	1105.69 ± 130.6	1117.4 ± 138.14	1093.97 ± 123.05
Total GM vol. (cm^3^)	559.21 ± 46.51	569.85 ± 69.1	579.84 ± 71.96	559.86 ± 66.24
Total CT (mm)	2.42 ± 0.1	2.4 ± 0.12	2.44 ± 0.11	2.37 ± 0.13
Total CV (cm^3^)	414.07 ± 35.34	421.14 ± 54.4	427.83 ± 56.24	414.45 ± 52.57
Total LGI	2.82 ± 0.09	2.83 ± 0.12	2.85 ± 0.13	2.82 ± 0.11
Motor circuit				
Subcortical GM (cm^3^)	51.5 ± 4.99	53.1 ± 6.26	54.98 ± 7.17	51.22 ± 5.36
CT (mm)	2.12 ± 0.10	2.11 ± 0.14	2.16 ± 0.14	2.07 ± 0.14
CV (cm^3^)	40.30 ± 3.5	41.20 ± 5.29	42.23 ± 4.89	40.17 ± 5.58
LGI	3.27 ± 0.16	3.30 ± 0.16	3.30 ± 0.18	3.30 ± 0.15

**Table 3:** Volume means and standard deviations of subcortical imaging-derived measures, for each group.

	HC	ET	LTG	HTG
Subcortical areas				
GM_Cerebellum (cm^3^)	94.60 ± 10.66	96.54 ± 11.84	98.02 ± 11.43	95.05 ± 12.29
lhCerebellumCor (cm^3^)	46.22 ± 5.26	47.23 ± 5.85	48.19 ± 5.80	46.27 ± 5.85
lhThalamus (cm^3^)	6.52 ± 0.94	7.04 ± 1.46	7.06 ± 1.29	7.01 ± 1.64
lhCaudate (cm^3^)	3.32 ± 0.51	3.50 ± 0.54	3.62 ± 0.56	3.38 ± 0.51
lhPutamen (cm^3^)	5.02 ± 0.83	5.13 ± 0.95	5.34 ± 0.98	4.92 ± 0.90
lhPallidum (cm^3^)	1.31 ± 0.24	1.38 ± 0.27	1.42 ± 0.27	1.34 ± 0.26
lhAccumbensa (cm^3^)	0.52 ± 0.13	0.52 ± 0.16	0.57 ± 0.16	0.47 ± 0.14
rhCerebellumCor (cm^3^)	48.37 ± 5.57	49.31 ± 6.11	49.84 ± 5.71	48.78 ± 6.56
rhThalamus (cm^3^)	6.10 ± 0.70	6.20 ± 0.99	6.56 ± 1.10	5.85 ± 0.72
rhCaudate (cm^3^)	3.51 ± 0.49	3.70 ± 0.58	3.81 ± 0.58	3.58 ± 0.56
rhPutamen (cm^3^)	4.75 ± 0.71	4.89 ± 0.78	4.96 ± 0.82	4.82 ± 0.75
rhPallidum (cm^3^)	1.47 ± 0.23	1.56 ± 0.24	1.51 ± 0.18	1.61 ± 0.27
rhAccumbensa (cm^3^)	0.52 ± 0.10	0.53 ± 0.14	0.57 ± 0.15	0.50 ± 0.12

**Table 4:** Between-group comparisons in whole-brain and motor circuit imaging-derived measures: HC versus LTG, HC versus HTG, and HC versus ET.

	Between-group tests								
	HC vs. LTG			HC vs. HTG			HC vs. ET		
	*p*	pFDR	*t*	*p*	pFDR	*t*	*p*	pFDR	*t*
Parenchymal volume	0.49	0.97	−0.69	0.34	0.68	−0.97	0.30	0.81	−1.03
GM volume	0.83	0.97	−0.21	0.44	0.68	−0.78	0.54	0.81	−0.61
CT	0.61	0.97	0.52	0.63	0.68	0.50	0.54	0.81	0.61
CV	0.82	0.97	0.22	0.50	0.68	−0.68	0.76	0.81	−0.31
LGI	0.94	0.97	−0.08	0.56	0.68	−0.59	0.71	0.81	−0.36
GM volume motor	0.08	0.78	−1.75	0.48	0.68	−0.72	0.13	0.81	−1.52
CT motor	0.97	0.97	−0.04	0.67	0.68	0.44	0.80	0.81	0.25
CV motor	0.49	0.97	−0.70	0.69	0.68	−0.41	0.51	0.81	−0.66
LGI motor	0.71	0.97	0.37	0.13	0.68	−1.56	0.48	0.81	−0.70

Effect sizes (*t*-statistic) and *p*-values are given after covarying for age, sex, and ICV, together with *p*-values, uncorrected and *p*-values, corrected by FDR, significant values for pFDR <0.05 *.

**Table 5:** Between-group comparisons in subcortical areas imaging-derived measures: HC versus LTG, HC versus HTG, and HC versus ET.

	Between-group tests								
	HC vs. LTG			HC vs. HTG			HC vs. ET		
	*p*	pFDR	*t*	*p*	pFDR	*t*	*p*	pFDR	*t*
GM_Cerebellum	0.61	0.94	−0.51	0.73	0.91	−0.36	0.59	0.84	−0.54
lhCerebellumCor	0.52	0.94	−0.64	0.75	0.91	−0.32	0.56	0.84	−0.6
lhThalamus	0.12	0.38	−1.58	0.06	0.42	−1.91	0.04	0.53	−2.07
lhCaudate	0.09	0.38	−1.72	0.68	0.91	−0.41	0.20	0.64	−1.29
lhPutamen	0.67	0.94	−0.42	0.95	0.95	−0.07	0.77	0.84	−0.29
lhPallidum	0.08	0.38	−1.78	0.83	0.91	0.21	0.38	0.84	−0.89
lhAccumbensa	0.96	0.97	0.04	0.81	0.91	0.25	0.86	0.86	0.18
rhCerebellumCor	0.72	0.94	−0.36	0.70	0.91	−0.38	0.65	0.84	−0.46
rhThalamus	0.31	0.79	−1.03	0.62	0.91	0.49	0.74	0.84	−0.34
rhCaudate	0.08	0.38	−1.77	0.46	0.91	−0.75	0.14	0.64	−1.5
rhPutamen	0.87	0.94	0.17	0.23	0.91	−1.21	0.52	0.84	−0.65
rhPallidum	0.78	0.94	−0.27	0.06	0.42	−1.86	0.18	0.64	−1.36
rhAccumbensa	0.86	0.94	−0.18	0.40	0.91	−0.85	0.56	0.84	−0.59

Effect sizes, *t*, and *p*-values are given after covarying for age, sex, and ICV, together with p-values, uncorrected and *p*-values, corrected by FDR, significant values for pFDR <0.05 *.

**Figure 5:**
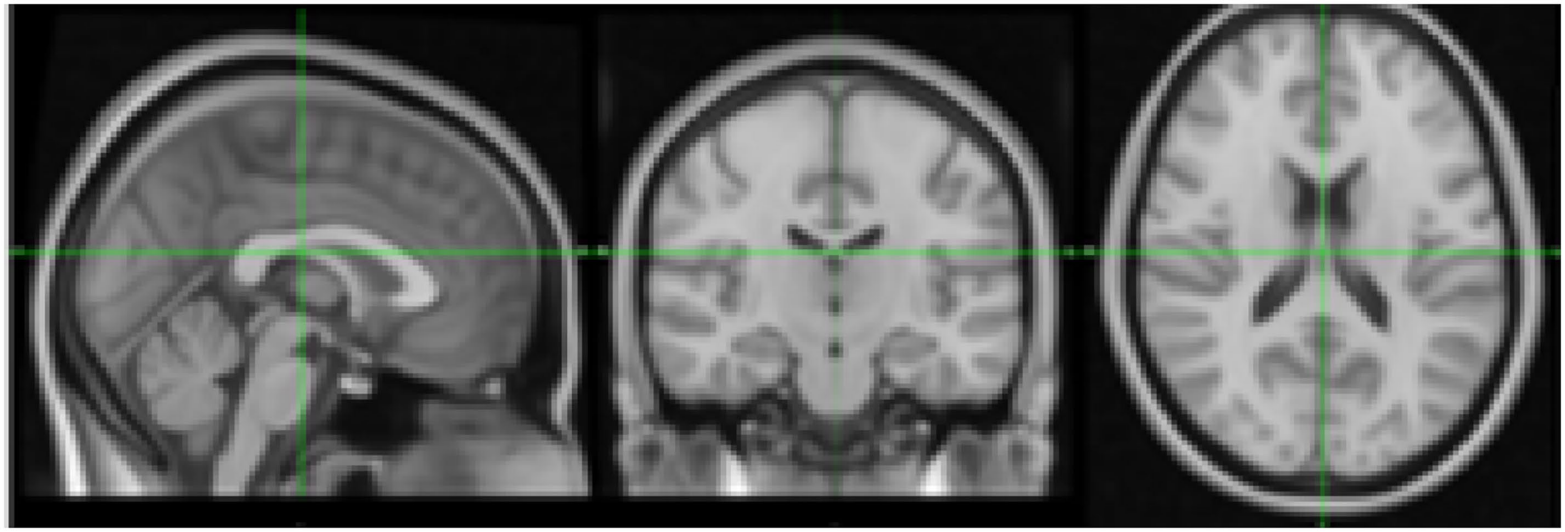
Non-significant regions (*p* < 0.05, TFCE-corrected) identified by between-group voxel-wise comparisons of GM volume for HC versus ET, HC versus LTG, and HC versus HTG.

Voxel-wise analyses of GM volume revealed no significant between-group differences (HC vs. ET, HC vs. LTG, or HC vs. HTG) after adjustment for age, sex, and ICV. Similarly, vertex-wise analyses of CV, CT, and LGI did not identify any significant differences across groups. Remarkably, both voxel-wise and vertex-wise analyses were additionally corrected using TFCE and Bonferroni correction, respectively, which resulted in the disappearance of all previously observed significant and trend-level effects in the cerebellum and the motor circuit. Overall, no robust regional alterations within the motor circuit or other cortical regions were detected after correction for multiple comparisons (Figure 5).

### 3.3 Within-patient group partial correlation with tremor severity

Partial correlations (and the corresponding *p*-values and pFDR) with tremor severity, TRS-A&B, controlling for age, sex, and ICV are shown in Tables 6, 7. Correlation analyses with tremor severity similarly demonstrated limited associations. Most structures showed weak and inconsistent relationships across groups. At the whole-brain level, partial correlations with tremor severity were non-significant (all uncorrected *p* > 0.05) for GM volume (ET: *p* = 0.62, *r* = −0.07; LTG: *p* = 0.82, *r* = −0.05; HTG: *p* = 0.41, *r* = −0.18), CT, and CV. LGI showed a sub-threshold uncorrected association in the HTG group (*p* = 0.06, uncorrected; *r* = −0.40). Parenchymal volume showed a nominally significant uncorrected correlation with tremor severity in the HTG group (*p* = 0.04, uncorrected; *r* = −0.44). Neither association survived the FDR correction. All p-values in this section are uncorrected unless explicitly stated otherwise; none of the correlations described survived correction for multiple comparisons. Partial correlations within the motor circuit (CT, CV, and LGI) were not significant. In contrast, subcortical analyses indicated subtle associations in ET involving the left and right pallidum. Modest negative correlations were observed between tremor severity and pallidal measures bilaterally in ET and HTG, as well as with cerebellar cortical measures in HTG patients (*r* ≈ −0.26 to −0.27). Conversely, positive correlations were identified for the right pallidum in HTG and ET groups, although these effects were small and statistically non-significant after correction. As in the previous analysis, none of these findings survived FDR correction (pFDR). Overall, the heterogeneity and low magnitude of these correlations argue against a simple linear relationship between tremor severity and regional subcortical volume loss.

**Table 6:** Within the ET group, partial correlations, r, with TRS-A&B controlling for age, sex, and ICV, together with *p*-values, uncorrected and *p*-values, corrected by FDR, significant values for pFDR <0.05 *.

	Partial correlation with tremor severity								
	LTG			HTG			ET		
	*p*	pFDR	*r*	*p*	pFDR	*r*	*p*	pFDR	*r*
Parenchymal volume	0.85	0.85	−0.04	0.04	0.29	−0.44	0.20	0.67	−0.19
GM volume	0.82	0.85	−0.05	0.41	0.79	−0.18	0.62	0.9	−0.07
CT	0.80	0.85	0.06	0.61	0.79	0.12	0.81	0.91	0.04
CV	0.84	0.85	−0.05	0.62	0.79	−0.11	0.96	0.96	−0.01
LGI	0.54	0.85	−0.14	0.06	0.29	−0.40	0.19	0.67	−0.19
GM volume motor	0.26	0.76	−0.25	0.70	0.79	−0.09	0.33	0.68	−0.14
CT motor	0.17	0.76	−0.3	0.83	0.83	−0.05	0.38	0.68	−0.13
CV motor	0.26	0.76	−0.25	0.44	0.79	−0.17	0.22	0.67	−0.18
LGI motor	0.34	0.76	−0.22	0.23	0.7	−0.26	0.70	0.9	−0.06

**Table 7:** Within the ET group, partial correlations, *r*, with TRS-A&B controlling for age, sex, and ICV, together with *p*-values, uncorrected and *p*-values, corrected by FDR, significant values for pFDR <0.05 *.

	Partial correlation with tremor severity								
	LTG			HTG			ET		
	*p*	pFDR	*r*	*p*	pFDR	*r*	*p*	pFDR	*r*
GM_Cerebellum	0.76	0.84	0.07	0.23	0.65	−0.27	0.18	0.51	−0.2
lhCerebellumCor	0.84	0.84	0.05	0.25	0.65	−0.26	0.15	0.51	−0.21
lhThalamus	0.82	0.84	0.05	0.88	0.98	0.03	0.91	0.96	−0.02
lhCaudate	0.23	0.84	−0.27	0.98	0.98	0.01	0.24	0.51	−0.18
lhPutamen	0.84	0.84	−0.05	0.64	0.98	−0.11	0.96	0.96	−0.01
lhPallidum	0.65	0.84	−0.1	0.22	0.65	−0.27	0.08	0.5	−0.26
lhAccumbensa	0.72	0.84	−0.08	0.82	0.98	−0.05	0.72	0.96	−0.05
rhCerebellumCor	0.70	0.84	0.09	0.23	0.65	−0.27	0.23	0.51	−0.18
rhThalamus	0.36	0.84	−0.21	0.71	0.98	−0.08	0.49	0.91	−0.1
rhCaudate	0.23	0.84	−0.27	0.45	0.84	0.17	0.71	0.96	−0.06
rhPutamen	0.34	0.84	−0.21	0.30	0.66	−0.23	0.89	0.96	0.02
rhPallidum	0.53	0.84	−0.14	0.16	0.65	0.31	0.06	0.5	0.28
rhAccumbensa	0.42	0.84	−0.18	0.90	0.98	−0.03	0.96	0.96	0.01

## 4 Discussion

The primary focus of this study was the accrual of neuroimaging evidence in support of two hypotheses that putatively explain the neuropathological mechanisms of ET, either as generalized degenerative process affecting widespread cerebral and cerebellar structures, or as a more selective degeneration restricted to the motor circuit, such as the cerebellum, thalamus, basal ganglia, and motor and premotor cortices.

Two complementary analytical strategies were employed. First, between-group analyses were performed to assess differences in regional brain measures between healthy controls (HC) and the ET groups. Second, correlation analyses were conducted to examine associations between tremor severity and ROI-derived measures across the ET groups. No significant changes were observed in grey matter metrics, including grey matter volume, cortical thickness, cortical volume, and cortical surface topology, at either the whole-brain level or within the predefined motor circuit (Tables 4–7). Subtle differences were observed in the uncorrected analyses but did not survive correction (Figure 4). Moreover, neither voxel-wise nor vertex-wise analyses revealed any significant group differences after correction for multiple comparisons (Figure 5). Three ET groups were examined, the full ET cohort comprising all patients, together with two subgroups stratified by tremor severity, the low tremor group (LTG), and the high tremor group (HTG).

Figure 3 illustrates how parenchymal volume is initially within the range of HC values, reducing as tremor severity increases, only exceeding one standard deviation of the HC distribution when TRS-A&B is in the range 30–35. Parenchymal volume was measured by the mask identifying the brain and takes no account of the spatial distribution of grey or other structural metrics. It therefore indicates that, as well as reductions in GM volume in the motor cortex and limited regions of the frontal cortex, ET may be associated with a broader pattern of generalized atrophy that becomes more pronounced as tremor intensity increases.

However, the between-group analyses did not identify robust structural brain differences at either global or motor circuit levels. Although minor variations in motor-related grey matter and gyrification were observed, measures of cortical morphology, including grey matter volume, cortical thickness, cortical volume, and gyrification, did not show significant between-group effects, suggesting no clear evidence of macroscopic cortical structural differences within the sensitivity of the present approach. At the subcortical level, no significant differences were detected in cerebellar, basal ganglia, or thalamic structures after correction for multiple comparisons. Exploratory analyses suggested only very subtle and inconsistent patterns in thalamic and striatal regions, without stable or reproducible effects across analyses. Cerebellar measures similarly did not show reliable structural differences or meaningful associations with tremor severity. Regarding the correlation analyses, a number of uncorrected trends were observed, although these were generally subtle, variable, and not fully consistent across measures or groups. These included weak associations between tremor severity and global measures, such as parenchymal volume and gyrification, together with exploratory effects involving several basal ganglia structures. However, these findings did not survive correction and should be interpreted cautiously as exploratory rather than definitive.

Overall, associations between structural measures and tremor severity were weak and inconsistent, without evidence of a clear or reproducible relationship. Moreover, the results suggest that ET is not associated with detectable structural brain differences in this cohort, and that any potential effects are likely to be subtle and below the resolution of the current morphometric approach. More sensitive or multimodal imaging methods may be required to further investigate potential network-level alterations.

What also emerges from these results is that the group of patients with low-intensity tremor (LTG) presents with symptoms without observable structural brain changes. HC versus LTG comparisons are non-significant throughout the analyses. With increasing tremor severity, some analyses show small and inconsistent trends in grey matter and cortical morphology, mainly in motor-related regions. However, these effects are weak and do not survive correction for multiple comparisons. Similar non-significant trends are observed in global brain measures, with no consistent pattern across analyses. The findings do not support a robust association between tremor severity and structural brain alterations, and any observed effects appear subtle and variable. Tremor is a common feature across many movement disorders.

The neuroimaging evidence to date of structural changes to the brain underlying this symptom has been summarized as abnormalities of the cerebello–thalamo–cortical network (Ortega-Robles and Arias-Carrión, 2025, 2025, 2025). While atypical oscillatory behavior within this network has been observed (Helmich et al., 2013), the precise origin of this effect and the mechanism that drives it remain obscure. This network overlaps with the motor circuit examined in the present study. In the high-tremor group (HTG), whole-brain voxel-wise analyses did not identify statistically significant grey matter differences after correction for multiple comparisons. Prior to correction, spatially distributed sub-threshold effects were observed, including exploratory trends in components of the motor circuit and the cerebellum; however, these were small, inconsistent across analyses, and did not survive FDR or TFCE correction. They are therefore reported solely for transparency and for hypothesis generation in future, larger studies. They do not constitute evidence of cerebellar structural alteration in ET.

Thus, these exploratory observations were not robust or consistent across analyses and remain exploratory in nature. Overall, there was no clear or reproducible evidence of a distinct pattern of structural changes within or beyond the motor circuit. Unfortunately, the hypothesis remains speculative and will require validation in cohorts with more comprehensive neuropsychological assessments.

Structural neuroimaging findings in essential tremor (ET) have shown variability across studies (Sharifi et al., 2014). New challenges and trends are appearing in ET research, particularly in the areas of early detection, precision diagnostics, personalized treatments, discovery of aetiologies that cause tremor, and experimental therapeutic trials (Lopez-de-Ipina et al., 2018; Lopez-de-Ipina et al., 2021; Erro et al., 2022; McGurrin et al., 2022; Otero et al., 2022; Gao et al., 2023). ET is a phenotypically heterogeneous disease (Bhatia et al., 2018; Louis, 2018) and can therefore be difficult to establish the degree of progression from clinical examination alone. Indeed, a new, albeit controversial ET-plus category has recently been suggested (Lenka and Jankovic, 2021) for patients with additional soft neurological signs, further complicating the ET diagnostic landscape.

Understanding the relationship between the severity of the key symptom and neuroimaging-derived biomarkers could provide valuable information for patient management and intervention. As noted above, prior neuroimaging studies have provided evidence of the role of the cerebellum in ET (Benito-León et al., 2009; Babij et al., 2013; Cerasa et al., 2009; Shin et al., 2016; van den Berg and Helmich, 2021), although some structural imaging studies show more widespread alterations (Sharifi et al., 2014; Benito-León et al., 2009). Both findings were also observed in the present study prior to correction. Notably, some studies did not find changes in the volume of the GM in ET patients, while others found both increases and decreases in GM (Sharifi et al., 2014). Our results suggest that effect sizes related to brain structures are small, and only studies with large samples or samples proportionately enriched with the most severely affected patients will be able to demonstrate significant neuroanatomical alterations using current imaging technology.

In this study, we applied well-established, robust structural neuroimaging approaches to characterize and identify the structural changes involved in ET. The study included patients with a wide range of tremor intensities, allowing an overview of the changes that occur in brain structure as the disease progresses, but also reducing statistical power by increasing variance. We tested two specific planned hypotheses using standard neuroanatomical atlases to define the motor circuit and the whole brain, and applied corrections for multiple comparisons to mitigate against false positives. The results of between-group and within-group analyses were consistent with each other and in line with the general direction of the previous literature, while providing additional insight into subtle associations between tremor severity and motor circuit structure. Specifically, no association between tremor severity and regional brain volume survived correction for multiple comparisons. Uncorrected exploratory trends in the direction of modest reductions are noted for hypothesis generation purposes only and must not be interpreted as evidence of a structural correlate of tremor severity without replication in adequately powered studies.

This study has some limitations. First, the wide range of tremor levels in the sample was selected at the expense of sample size (*N* = 50 patients), limiting the power and generalizability, and hence the results must be replicated and extended in larger cohorts specifically designed for this purpose. However, the selection of hypothesis-driven biomarkers provides a robust experimental framework that adds value, together with the novel methodology and results. Although we divided the patient cohort into LTG and HTG and drew inferences from it about disease progression, these results require confirmation with a longitudinally designed study. Additionally, head motion, a common feature in patients with chronic or advanced ET, represented a potential confound. HTG patients had increased head movement, that is, Euler number, relative to HC. Visual inspection of the T1-weighted images did not uncover any adverse changes in image quality in the HTG group; however, its effect on bias and variance cannot be discounted. The voxel-wise models were regressed with the Euler number as a covariate without changing the results.

Future studies should acquire a wider range of clinical, familial, and environmental metrics to explore the factors influencing the etiology of ET and its progression as a contribution to the evidence for preventative advice or treatments. Issues related to the diagnosis and classification of tremor syndromes, such as the presence of family history or response to alcohol, are not currently included in the diagnostic criteria for ET, and it would be interesting to see whether these two features are predictive of its evolution or indicative of differential neuropathology. Other MRI sequences, particularly diffusion imaging, which depicts white matter integrity, are available for future studies of the corticospinal tract and corpus callosum, which provide anatomical connectivity to the motor circuit (Bullock et al., 2022). Together with post-mortem studies, understanding more about the anatomy of ET in the motor circuit and beyond will be key to further improvement of patient outcomes.

## 5 Conclusion

Essential tremor is the most common movement disorder, and early diagnosis, monitoring, and appropriate management of the disease are essential for optimizing clinical outcomes. Mechanistic models based on non-invasive imaging data are powerful tools to understand and investigate potential treatments and symptom management. In this work, the analysis of ET severity in relation to brain structure revealed only subtle structural differences, mainly within the motor circuit, which did not remain significant after correction for multiple comparisons. These findings are consistent with previous literature, suggesting that structural MRI abnormalities in ET are subtle and heterogeneous across studies that are likely only detectable on T1-weighted MRI when the symptom becomes severe (TRS-A&B > 30). Although our study was not designed to address disease progression directly, the observed patterns may be compatible with the hypothesis of progressive structural alterations in ET, particularly involving the motor circuit.

## Data Availability

The datasets generated by and/or analysed during the current study are not publicly available due to ethics and privacy requirements, but they are available from the corresponding author upon reasonable request. All the code is open source upon reasonable request.
